# Microstructure and Mechanical Properties of Al_2_O_3_/Er_3_Al_5_O_12_ Binary Eutectic Ceramic Prepared by Bridgman Method

**DOI:** 10.3390/ma11040534

**Published:** 2018-03-30

**Authors:** Caiyu Song, Shunheng Wang, Juncheng Liu, Shuoyan Zhai

**Affiliations:** 1 School of Materials Science and Engineering, Tianjin Polytechnic University, Tianjin 300387, China; 1530021076@stu.tjpu.edu.cn (C.S.); 1720021049@stu.tjpu.edu.cn (S.Z.); 2 School of Materials Science and Engineering, Shandong University of Technology, Zibo 255049, China; 16509140461@stumail.sdut.edu.cn

**Keywords:** Al_2_O_3_/EAG ceramic, directional solidification, Bridgman method, growth rate, mechanical properties, toughening mechanism

## Abstract

Directionally solidified Al_2_O_3_/Er_3_Al_5_O_12_ (EAG) eutectic ceramic was prepared via vertical Bridgman method with high-frequency induction heating. The effects of the growth rate on the microstructure and mechanical properties of the solidified ceramic were investigated. The experimental results showed that there were no pores or amorphous phases in the directionally solidified Al_2_O_3_/EAG eutectic ceramic. Al_2_O_3_ phase was embedded in the EAG matrix phase, and the two phases were intertwined with each other to form a typical binary eutectic “hieroglyphic” structure. With the increase of growth rate, the phase size and spacing of the solidified Al_2_O_3_/EAG ceramic both decreased, and the growth rate and phase spacing satisfied the *λ*^2^*v* ≈ 60 formula of Jackson-Hunt theory. The cross section microstructure of the solidified ceramic always exhibited an irregular eutectic growth, while the longitudinal section microstructure presented a directional growth. The mechanical properties of the solidified ceramic gradually increased with the increase of growth rate, and the maximum hardness and fracture toughness could reach 21.57 GPa and 2.98 MPa·m^1/2^ respectively. It was considered that the crack deflection and branching could enhance the toughness of the solidified ceramic effectively.

## 1. Introduction

In recent years, the aerospace industry has put forward more stringent requirements on the high-temperature performance and safety of aircraft engine core components (turbine blades and the like). At present, Nickel-based and cobalt-based single crystal alloys are the most widely used turbine blade materials in the aerospace field, of which the highest working temperature can be up to 1100 °C. However, their mechanical properties above 1100 °C will be significantly degraded [1], and their high temperature stability and safety cannot meet the aviation needs. SiC/SiC [2], C/C [3,4] and Si_3_N_4_ [5] non-oxide composites possess higher melting point, but they are easily oxidized in the ultra-high temperature air environment, or at least their anti-oxidation ability is far from meeting the needs of aero engines, which leads to the sharp deterioration of their mechanical properties.

For engines with a thrust-weight ratio of 10 or more, the temperature of turbine’s intake port has now exceeded 1600 °C [6], so the demand for ultra-high temperature materials is even more pressing. At the end of the 20th century, A. Sayir [7] reported a promising ultra-high temperature material—Al_2_O_3_/EAG oxide eutectic composite ceramic, which has great potential for long-term operation in a high temperature environment above 1600 °C [7,8,9]. Compared with sintered ceramics, the oxide eutectic composite ceramic has compact structure but no grain boundaries or other amorphous phases, thus endowing it with excellent high temperature resistance, high strength and outstanding creep resistance [10,11,12]. In addition, the Al_2_O_3_/EAG eutectic ceramic can emit narrow and sharp radiation peak at the wavelength of 1.5 µm [13], which can also be used as a functional material with potential value for high temperature and oxidation resistant environments in aerospace. Although the brittleness may limit the service life and ultra-high temperature mechanical properties of oxide ceramics, the vertical Bridgman directional solidification technology can improve the long-term stability of oxide eutectic ceramics and enhance their mechanical properties at the high temperature. The vertical Bridgman method has several advantages, such as simple operation, low cost, and high possibility of preparing large volume samples. Nakagawa and Waku [13] prepared Al_2_O_3_/EAG eutectic ceramic via this method. However, due to the low growth rate (5 mm/h) and small temperature gradient, the phase size and spacing in its microstructure are larger and the microstructure contains cellular or granular structure, which limits the further improvement of its mechanical properties. Therefore, it is particularly important to increase the temperature gradient and growth rate of the eutectic ceramic so as to improve its microstructure and enhance the mechanical properties. If it is ensured that the melt can form stable eutectic structure in the eutectic zone, the large temperature gradient and high growth rate can promote the melt convection and solute diffusion in front of the solid-liquid interface, thus increasing the solidification rate and refining the eutectic structure. As a result, the mechanical properties of the materials can be improved significantly [14].

In this study, directionally solidified Al_2_O_3_/Er_3_Al_5_O_12_ (EAG) eutectic ceramic was prepared via vertical Bridgman method with high-frequency induction heating, which provided a large solid-liquid interface temperature gradient and a high growth rate for the eutectic growth. Then, the effects of growth rate (*v*, 0–24 mm/h) on the microstructure and mechanical properties of Al_2_O_3_/EAG eutectic ceramic were investigated.

## 2. Experimental

### 2.1. Preparation of the Sintered Al_2_O_3_/EAG Ceramic

High-purity Al_2_O_3_ powder (99.99%) and Er_2_O_3_ (99.99%) powders (Tianjin Fengchuan Chemical Reagents Technology Co., Ltd., Tianjin, China) were used as the raw materials, and they were weighed according to the eutectic mole fraction of Al_2_O_3_:Er_2_O_3_ = 81:19 in the phase diagram [15]. The raw materials were added to a ball mill jar with anhydrous ethanol as dispersant, and the grinding ball is made of alumina. The rotating speed of the planetary mill with alumina ball was adjusted to 350 r/min. The materials were milled in the ball mill jar for 4 h and then dried in a drying oven at 70 °C for 24 h. After that, about 5 wt % of PVA binder was added into the power and the mixed powder was pressed into Φ10 mm × 15 mm mold under the pressure of 10 MPa to obtain a green rod. All rods were dried in a constant temperature oven at 200 °C for 24 h, and finally sintered for 2 h at 1500 °C in a high-temperature furnace to obtain the sintered ceramic rods.

### 2.2. Preparation of the Solidified Al_2_O_3_/EAG Ceramic via Bridgman Method

The prepared sintered ceramic was placed in a tungsten crucible with the size of Φ10 mm × 150 mm. When the vacuum was evacuated to below 1 × 10^−2^ Pa, the temperature of crucible wall was heated to 2200 °C by multi-turn cylindrical coils and high frequency induction power supply. After incubation for 30 min, the sintered ceramic in the tungsten crucible was melted completely. Then the crucible vertically descended at a rate of 6 mm/h, 12 mm/h, 18 mm/h and 24 mm/h respectively. Thus, the sintered ceramic in the crucible solidified and crystallized from bottom to top to achieve the directional growth of Al_2_O_3_/EAG ceramic.

### 2.3. Characterization

**Figure 1:**
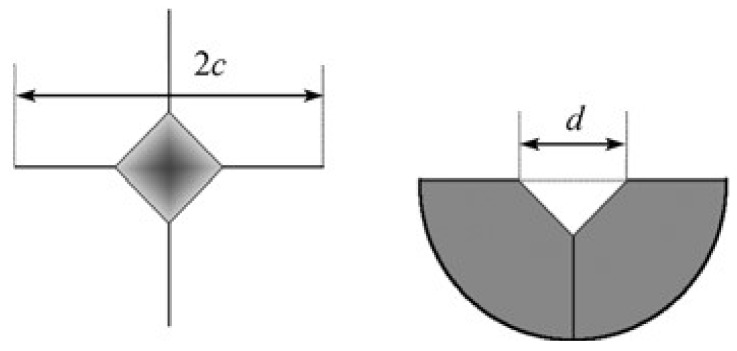
Schematic diagram of Vickers-Hardness indentation.

The sample phase compositions were determined with X-ray diffraction (XRD, D/MAX-2500, Rigaku, Tokyo, Japan, Cu-K*α*1, *λ* = 0.15405 nm) with Energy Dispersive Spectrometer (EDS). In the experiment, the 2 theta angle was 10~80 degrees, the acceleration voltage was 40 kV, and the step length was 0.02. The sample was sliced with a diamond wire, and the microstructure of the ceramics was observed by S-4800 cold field emission scanning electron microscope (FSEM). SISC.IAS V8.0 metallographic image analysis software was used to analyze and calculate the volume fraction of each phase in the solidified ceramic. The hardness of the ceramics was measured by the Vickers-Hardness micro-hardness tester (HVS-5, Yantai Huayin Experimental Instrument Co. Ltd., Yantai, China), and the fracture toughness of the solidified ceramic was calculated by the indentation method. Figure 1 shows the schematic diagram of Vickers-Hardness indentation [16]. A 9.8 N load was applied and lasted for 15 s to obtain the hardness value. Such operation was repeated 10 times at different positions for one sample, and the average value was taken as the final hardness of the sample. Then, the fracture toughness of the solidified ceramic was calculated according to the hardness value and indentation crack length.

## 3. Results and Discussion

### 3.1. Phase Analysis

**Figure 2:**
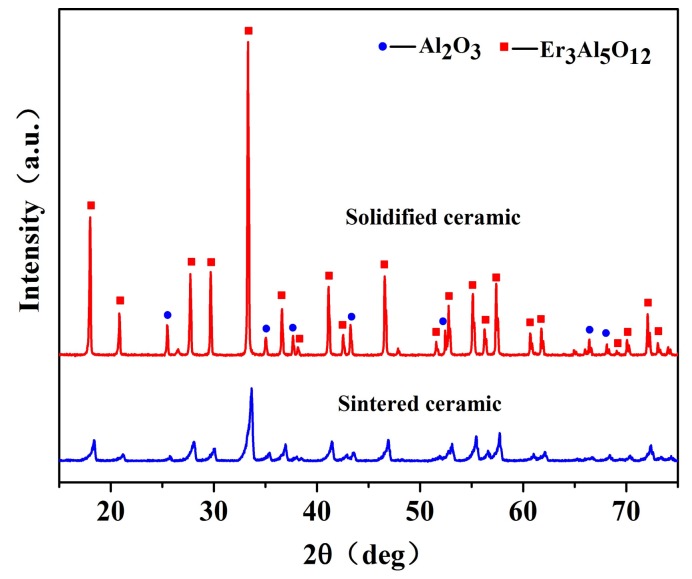
XRD patterns of the solidified Al_2_O_3_/EAG ceramic (18 mm/h) and the sintered one.

The Al_2_O_3_/EAG sintered ceramic and solidified ceramic were ground into powder respectively, and their XRD patterns are shown in Figure 2.

As can be seen from Figure 2, both the solidified ceramic and the sintered one are consisted of Al_2_O_3_ phase and EAG phase, and there are no amorphous phases. These results are consistent with the results of Su [17]. Compared with the sintered ceramic, the diffraction peaks of the solidified ceramic become sharp and narrow, and their intensity also increases greatly. This means that the crystallinity and the crystal integrity of the solidified ceramic are improved significantly.

### 3.2. Microstructure

**Figure 3:**
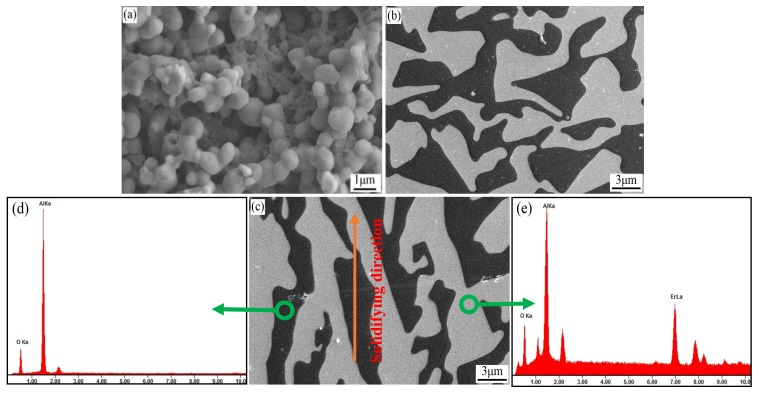
Microtopography of the sintered ceramic and Bridgman solidified ceramic (12 mm/h): (**a**) the sintered ceramic; (**b**) cross section of the solidified ceramic; (**c**) longitudinal section of the solidified ceramic; (**d**,**e**) EDS spectra of the solidified ceramic.

Figure 3 shows the microstructures of the sintered and solidified Al_2_O_3_/EAG ceramics.

It is clear that there are pores and grain boundaries in the sintered ceramic (Figure 3a), whereas no pores or grain boundaries can be observed in the solidified ceramic (Figure 3b,c). From Figure 3b,c, it can be seen that both the cross section and longitudinal section microstructures of the solidified ceramic present irregular eutectic structures; the longitudinal section microstructure shows a clear growth direction near to the solidifying direction. From the EDS spectra (Figure 3d,e), the black region in Figure 3c was mainly composed of Al and O, and the gray region is mainly composed of Al, O and Er. Combined with XRD analysis results, it is confirmed that the solidified ceramic is indeed composed of two phases of Al_2_O_3_ (black) and EAG (gray). The two phases are intertwined and coupled to each other to form a typical binary eutectic “hieroglyph” structure [18,19].

The volume fraction of each phase was calculated by SISC.IAS V8.0 metallographic image analysis software. The results showed that the volume fraction of Al_2_O_3_ phase was about 40 ± 3%, which was consistent with the theoretical value of 42.5% [20,21].

### 3.3. Effects of Growth Rate on the Microstructure of the Solidified Ceramic

In the ceramic growth process, the temperature gradient and growth rate are the main factors affecting the eutectic structure. However, the growth rate plays a decisive role once the temperature gradient is determined [17,22]. During the preparation of eutectic materials, if the eutectic structure can grow steadily in the eutectic symbiotic region, a specific supercooling degree is required. If the supercooling degree is not satisfied, cellular or dendritic eutectic structure may dominate, thus reducing the mechanical properties of the prepared materials. When the eutectic structure grows steadily in the eutectic region, the supercooling degree (*ΔT*), the temperature gradient (*G*), the growth rate (*v*) and the solute diffusion rate *(D*) need to satisfy a certain relationship, as shown in Formula (1) [14].

*ΔT* = *GD*/*v* + *Kv^W^* (1)

where *K* and *W* are constants relating to the nature of the eutectic composition. It can be seen from Formula (1) that once *G* and *D* are determined, the corresponding *ΔT* can be obtained for a given *v.*

**Figure 4:**
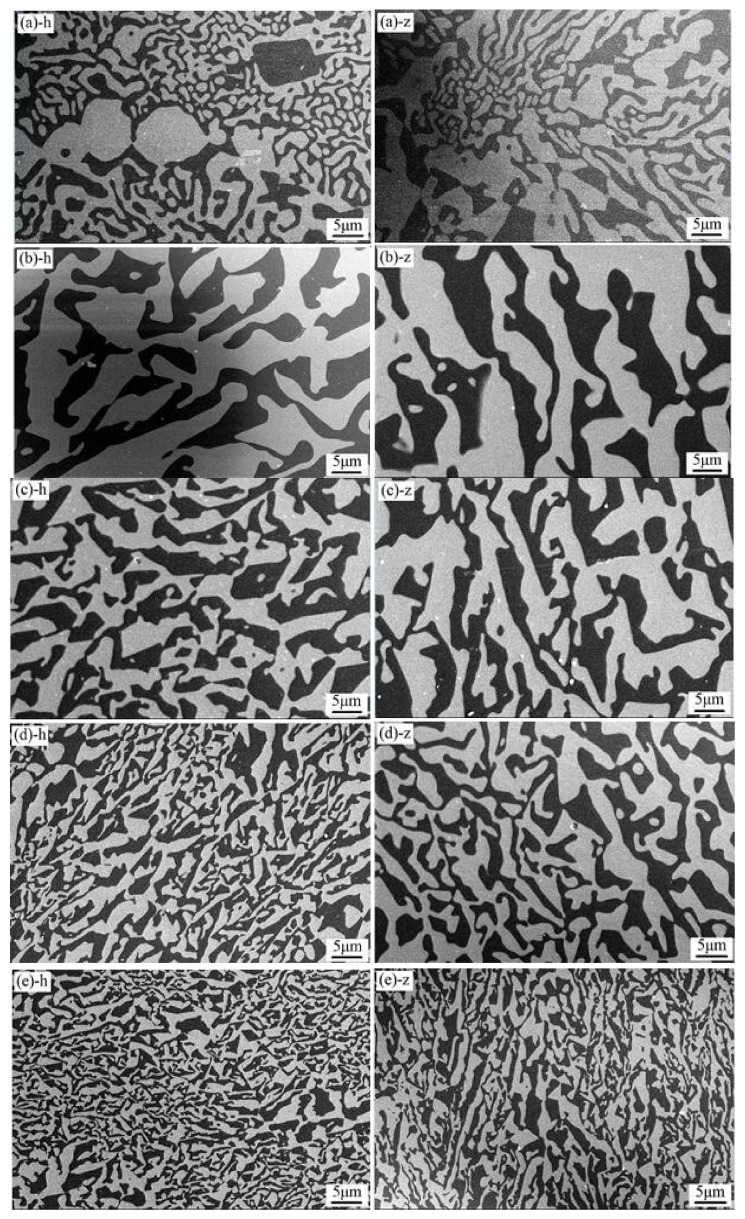
Effects of growth rate on the microstructure of the solidified ceramic prepared at different growth rates: (**a**) *v* = 0 mm/h; (**b**) *v* = 6 mm/h; (**c**) *v* = 12 mm/h; (**d**) *v* = 18 mm/h; (**e**) *v* = 24 mm/h (h stands for the cross section and z stands for the longitudinal section).

Figure 4 shows the effects of growth rate on the microstructure of the solidified ceramic. As can be seen, the shape and size of either EAG or Al_2_O_3_ phase are very inhomogeneous when the growth rate is 0 mm/h (without walking), and the longitudinal section does not show any directional growth.

With the increase of growth rate from 6 mm/h to 24 mm/h, the eutectic spacing and the phase size gradually decrease, and no cell or dendritic eutectic structure is formed. Due to the increase of growth rate, the front temperature gradient and solidification rate at the solid-liquid interface of the solidified ceramic both increase, which results in the refinement of its microstructure [16].

Due to the irregular eutectic structure of the solidified Al_2_O_3_/EAG ceramic and the entangled distribution of the two phases, the measurement of eutectic spacing is fairly complicated. Therefore, the average eutectic spacing of the two phases is expressed by the interface length per unit area of Al_2_O_3_ phase and EAG phase, as shown in Formula (2) [23]:

*Λ* = 2*a*^2^/*l* (2)

where *a* is the length of the SEM square image, and *l* is the total interface length of the two phases. This method has strong objectivity for measuring the average eutectic spacing. Mizutani et al. [23] ever used this method to measure the phase spacing of Al_2_O_3_/YAG irregular eutectic microstructure, and it was considered that the results were consistent with those from the conventional method. The results from Formula (2) are shown in Table 1. It can be found that *λ*^2^*v* ≈ 60 (constant), satisfying Jackson-Hunt theory [24].

### 3.4. Hardness and Fracture Toughness of the Solidified Al_2_O_3_/EAG Ceramic

**Figure 5:**
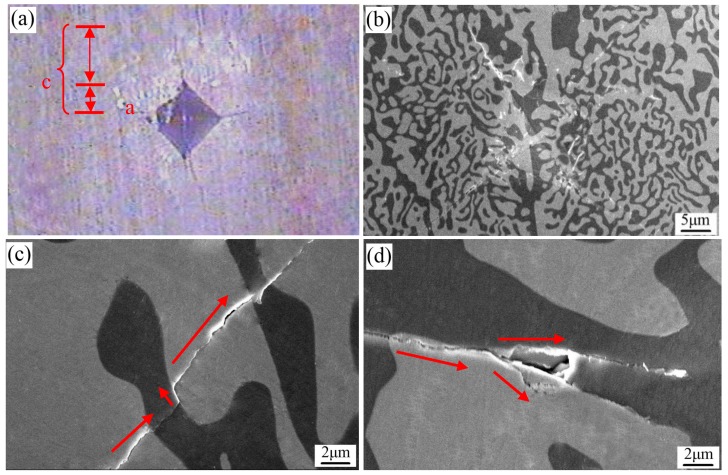
(**a**) the metallographic micro-indentation morphology; (**b**) SEM micro-indentation morphology; (**c**) the crack deflection; (**d**) the crack branching.

Figure 5 shows the indentation and crack morphology of the solidified Al_2_O_3_/EAG binary eutectic ceramic. It can be identified that the indentation crack belongs to the Median crack (*c/a* > 1.25) [25]. Hence, the fracture toughness [26] can be calculated by the following formulas:

(3) $$K_{IC}=0.016{\left(\frac{E}{H_{V}}\right)}^{\frac{1}{2}}Pc^{-\frac{3}{2}}$$

where *P* is the load (9.8 N), *E* is the elastic modulus (about 311 GPa [21]), *c* is the crack half-length, *d* is the average of the indentation diagonal length, and *H_V_* is the hardness, which is read directly by the hardness tester. According to Formula (3), the fracture toughness of the sintered ceramic and the solidified one were calculated.

**Figure 6:**
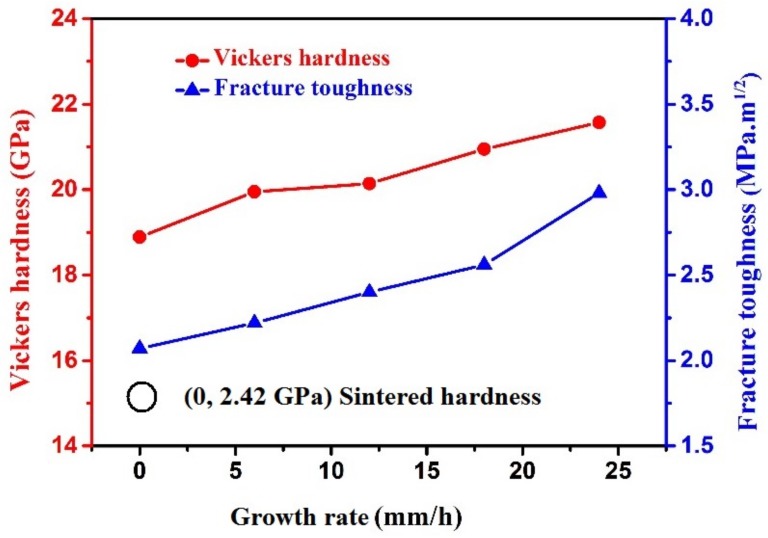
Effects of growth rate on hardness and fracture toughness of the solidified Al_2_O_3_/EAG ceramic.

Figure 6 shows the effects of growth rate on the hardness and fracture toughness of the solidified Al_2_O_3_/EAG ceramic. Both the hardness and fracture toughness of the solidified Al_2_O_3_/EAG ceramic gradually increase with the increase of growth rate. When the growth rate *v* = 24 mm/h, the hardness and fracture toughness of the solidified Al_2_O_3_/EAG ceramic reach the maximum values of 21.57 GPa and 2.98 MPa·m^1/2^ respectively, which are higher than those reported by Özerdem [27] or Mesa [19]. From Figure 6, the maximum hardness of the solidified ceramic is about 8.9 times that of the sintered ceramic with the same composition. This is mainly related to the absence of grain boundaries and pores in the microstructure of the sintered ceramic, because pores and grain boundaries play a crucial role in degradation of the mechanical properties of ceramic materials [28]. Here, the relative density of the sintered ceramic is only about 82.9%. In addition, the two phases of Al_2_O_3_ and EAG are intertwined with each other and present a uniform distribution, which can also affect the mechanical properties of the solidified ceramic to some extent.

As can be seen from Figure 5c (as indicated by the red arrows), crack deflection occurs twice at the interface of Al_2_O_3_ and EAG phases during the propagation process. It is well known that crack deflection is beneficial to the residual stress of the wave and can effectively reduce the driving force of crack propagation. Crack propagation in this way can improve the toughness of brittle solid materials to a certain degree [29]. In addition, it can be seen from Figure 5d that crack branches along the phase boundary as it propagates (as indicated by the red arrows). The crack branching can effectively prevent crack propagation and reduce crack tip stress, which also enhances the toughness of the solidified ceramic. Therefore, there are two reasons for the enhanced fracture toughness of the solidified Al_2_O_3_/EAG ceramic with the increase of growth rate. Firstly, the microstructure of the solidified Al_2_O_3_/EAG ceramic is very compact, and the phase spacing becomes smaller with the increase of growth rate, which increases the total phase boundary interface significantly and produces more tortuous phase interface. When plastic deformation occurs, the interactions between the fine phases and the cracks are enhanced [16], which can reduce stress concentration, increase plastic deformation capacity and impede crack expansion, thus improving the fracture toughness [30]. Secondly, the crack deflection and branching in the microstructure of the solidified ceramic can effectively reduce the driving force of crack propagation, and thus improve the toughness of the solidified ceramic.

## 4. Conclusions

In summary, directionally solidified Al_2_O_3_/Er_3_Al_5_O_12_ (EAG) eutectic ceramic was prepared via vertical Bridgman method with high-frequency induction heating. The effects of the directionally solidification process and its growth rate on the microstructure and mechanical properties of the eutectic ceramic were investigated.

(1) The sintered ceramic was composed of polycrystalline grains, which contain pores and grain boundaries. While there were no pores and grain boundaries in the directionally solidified Al_2_O_3_/EAG eutectic ceramic, where Al_2_O_3_ phase was embedded in the EAG matrix phase, and the two phases were intertwined with each other to form a typical binary eutectic “hieroglyphic” structure. The cross section microstructure of the solidified ceramic always exhibited an irregular eutectic growth, while the longitudinal section microstructure presented a directional growth.
(2) The maximum hardness and fracture toughness of the directionally solidified ceramic could reach 21.57 GPa and 2.98 MPa·m^1/2^ at the growth rate of 24 mm/h, respectively. The former is 8.9 times that of the sintered ceramic. The main reason could be attributed to the absence of grain boundaries and pores in the microstructure of the sintered ceramic, playing a crucial role in degradation of the mechanical properties of ceramic materials. And the crack deflection and the branching are the main toughening mechanisms of the solidified ceramic.
(3) The mechanical properties of the solidified ceramic gradually increased with the increase of growth rate. This could be attributed mainly to the refinement of microstructures. With the increase of growth rate, the phase size and spacing of the solidified Al_2_O_3_/EAG ceramic both decreased, and the growth rate and phase spacing satisfied the *λ*^2^*v* ≈ 60 (constant) formula of Jackson-Hunt theory. The finer the microstructure, the more significant the toughening effects of the crack deflection and the branching.

## Figures and Tables

**Table 1:** Effects of the growth rate on eutectic spacing (*λ*) of the solidified ceramic.

*v* (μm/s)	*λ* (μm)	*λ*^2^*v*
1.67	6.09	61.94
3.33	4.28	61.00
5.00	4.28	59.51
6.67	3.00	60.03
